# A case of diabetic foot with severe vascular disease in an elderly no-option patient undergoing lower limb arterialization: a clinical case report

**DOI:** 10.1007/s11739-025-04106-y

**Published:** 2025-08-29

**Authors:** Adriano Massacesi, Gioia Palmonella, Cristina Gatti, Michela Fichetti, Enrico Paci

**Affiliations:** 1 Diabetology and Metabolic Diseases, Diabetic Foot Center, IRCCS INRCA, Ancona, Italy; 2 Diagnostic Imaging, Clinical and Interventional Radiology, IRCCS INRCA, Ancona, Italy

**Keywords:** Lower limb arterialization, Peripheral artery disease, Gangrene, Transcutaneous oximetry, WIFI classification

## Abstract

This clinical case report describes an 80-year-old no-option diabetic patient with chronic limb-threatening ischemia and severe vascular disease. The patient presented with wet gangrene and underwent an unsuccessful revascularization attempt, followed by minor amputation complicated by poor wound healing. Due to persistent ischemia and surgical dehiscence, the patient underwent a transcatheter deep venous arterialization procedure, resulting in improved perfusion, wound healing, and limb salvage. This case highlights the potential of venous arterialization as a limb-saving option in carefully selected elderly patients, emphasizing the importance of a multidisciplinary and individualized approach.

## Case presentation

**Fig. 1:**
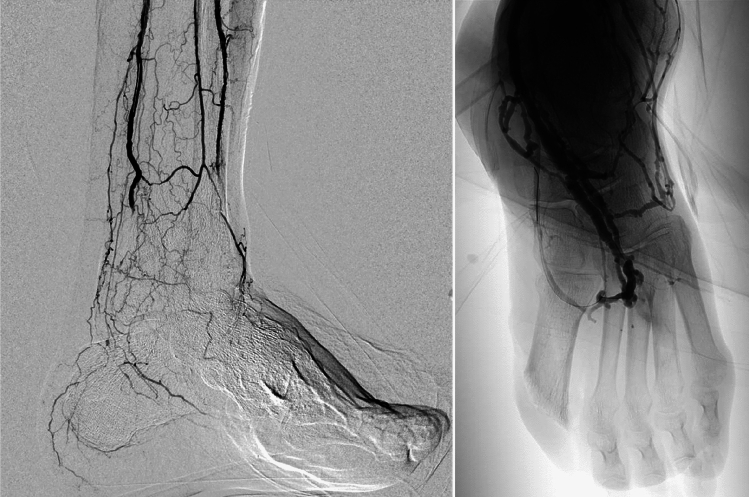
On the left, final arteriographic result after second percutaneous angioplasty, showing poor pedal circulation. On the right, optimal final arteriographic result after arterialization procedure

In January 2024, an 80-year-old man with multiple comorbidities (type 2 diabetes, neuropathy, AOCAI, hypertension, dyslipidemia, rheumatoid arthritis) was referred to our Diabetic Foot Clinic for wet gangrene of the first toe, with lymphangitis extending to the ankle, after failed antibiotic therapy for a first-ray ulcer. On admission, he had a cold, painful left foot. Labs showed neutrophilic leukocytosis (13,070/mm^3^), thrombocytosis, and elevated CRP (6.69 mg/dL). (Table 1). Broad-spectrum antibiotics (piperacillin/tazobactam + teicoplanin) were started with good response. TcPO₂ values were very low (D: 9 mmHg, M: 12 mmHg), ABI was 0.7 (PTA), with AT non-compressible. WIfI stage was 3-3-2 (Stage 4). Previous Doppler showed patent tibial and pedal arteries with calcified walls and indirect flow. Two percutaneous angioplasties failed. (Fig. 1) He underwent first-ray amputation complicated by flap necrosis. Considered a no-option limb, he was selected for venous endovascular arterialization of the posterior tibial vein, achieving excellent arteriographic outcome. (Fig. 1). Discharge therapy included enoxaparin, later replaced by rivaroxaban 2.5 mg and Aspirin 100 mg. TcPO₂ improved markedly at 3 and 5 months (up to 61 mmHg). Given high risk of re-ulceration, a transmetatarsal amputation with dermal matrix application was performed after 3 months. NPWT led to complete wound healing.

**Table 1:** Laboratory tests on admission to the department

Laboratory test	Values	Normal range
Hb (g/dl)	14.4	from 13,0 to 17,5 g/dl
WB (X10/3/microL)	13,070/mmc	from 4,00 to 10,00
NEUTROPHILES	8532/mmc	2,00–7,50 (X10/3/microL)
PLT (X10/3/microL)	448	from 140 to 400 (X10/3/microL)
CPR (mg/dl)	6,69	< 0,50 mg/dl
Creatinine (mg/dl)	0,8	From 0,70 to 1,20 mg/dl
PTT sec	40	Not in therapy 28–42 s
Pro-Calcitonin (ng/ml)	< 0.05	Absent < 0,05
PT (%)	88	Not in therapy from 70 to 120
INR	1,09	Not in therapy from 0,8 to 1,25

## Discussion

Arterial revascularization, either endovascular and/or surgical, is the standard procedure for the treatment of patients with lower limb ischemia [1].

In Europe, it is estimated that 40 million individuals are affected by peripheral artery disease (PAD), with a prevalence of approximately 5.3%, based on a population of 750 million inhabitants [2].

Despite the availability of numerous endovascular and surgical techniques for treating lower limb ischemia, a subset of patients is not eligible for revascularization procedures and is therefore classified as “no-option”.

In the absence of restored blood flow, chronic limb-threatening ischemia (CLTI) in no-option patients characterized by pain, non-healing wounds, and gangrene typically progresses to major amputation (above the ankle).

Major amputation due to CLTI is associated with a 50% one-year mortality rate in patients over 65 years old, with even higher mortality among those with concomitant cardiovascular diseases [3].

Transcatheter arterialization of deep veins is an endovascular revascularization technique used to treat no-option chronic limb-threatening ischemia.

When performed in the lower limbs, the procedure involves creating an arteriovenous fistula proximal to the diseased tibial arteries using a covered stent. Oxygenated blood is thus diverted from the tibial arteries into the tibial veins, bypassing the severely diseased arterial segment. The venous system is then utilized to deliver oxygenated arterial blood to the foot through the plantar veins, potentially avoiding major amputation and promoting wound healing [4].

Patients considered for the procedure must be carefully selected, as it is contraindicated under specific conditions. These include the absence of an adequate patent deep or superficial venous axis, which requires a target vein that is sufficiently patent and free of thrombosis. Recent or extensive deep vein thrombosis (DVT) precludes the use of veins for retrograde arterial perfusion. Uncontrolled systemic infection or sepsis significantly increases perioperative risk. Advanced, uncompensated heart failure (e.g., NYHA class IV) cannot tolerate the increased afterload caused by the arteriovenous shunt. Severe coagulopathy or active bleeding poses a high risk of hemorrhagic complications. Lastly, advanced malignancy with limited life expectancy renders the procedure futile.

In this paper, we reported a case of a geriatric patient with diabetic foot and a high risk of major limb amputation based on clinical presentation.

According to the WIfI classification (3-3-2), the patient’s overall stage of 4 corresponded to an estimated 1-year amputation risk of 20–50% [5].

Considering the high 5-year mortality rate associated with major amputation in elderly diabetic patients, lower limb arterialization proved to be a valuable option for preventing limb loss in this case.

In our patient, the procedure was technically successful, with progressive improvement in local clinical parameters and transcutaneous oximetry values.

In many cases, the arterialization procedure is repeated to perform additional interventions or surgical revisions aimed at optimizing blood flow or managing complications, or to carry out complementary procedures if the clinical condition requires it. In this case, the optimal transcutaneous oximetry values demonstrated the clinical success of the single procedure, making repetition unnecessary.

The postoperative course was uneventful and led to complete re-epithelialization of the surgical site, with satisfactory functional recovery and maintenance of autonomous ambulation with assistance devices.

This clinical case highlights how this procedure may represent a limb-salvage strategy for patients with no vascular options.

It should be noted, however, that despite being an elderly patient, he did not suffer from heart failure or have a high bleeding risk.

Therefore, careful consideration must be given to multimorbidity, which may expose patients to procedural failure, as well as the need for intensive antiplatelet/anticoagulant therapy to prevent stent reocclusion, this poses an already elevated risk in elderly individuals.

## Conclusion

This case report illustrates that arterialization in a diabetic no-option patient may serve as a valid therapeutic strategy to prevent major amputation, which would otherwise increase mortality risk within the first 5 years.

However, patient selection should follow a multidisciplinary evaluation. Candidates must not have terminal heart failure and should possess a suitable peripheral venous network. Additionally, the bleeding risk must be thoroughly assessed due to the need for intensive antiplatelet or anticoagulant therapy after the procedure.

This case emphasizes the importance of a multidisciplinary and personalized approach to managing unrevascularizable critical limb ischemia. Venous arterialization emerges as a promising salvage therapy in selected patients, offering the possibility of avoiding major amputation and improving quality of life, even in advanced age, while preserving walking ability.

## Abbreviations

ABI: Ankle-Brachial Index
WIfI: Wound, ischemia, foot infection
CRP: C-reactive protein
PLT: Platelets
D: Dorsal
M: Medial
AOCAI: Chronic obliterative arterial disease of the lower limbs
MRSA: Methicillin-resistant *Staphylococcus aureus*
NPWT: Negative pressure wound therapy

## Declarations

### Use of AI

The authors used ChatGPT (Opne AI, San Francisco, CA, USA) for language translation support. The final version was reviewed and verified by the authors.

## References

[1] Mazzolai L, Teixido-Tura G, Lanzi S et al (2024) 2024 ESC guidelines for the management of peripheral arterial and aortic diseases. Eur Heart J. 10.1093/EURHEARTJ/EHAE179 39210722 10.1093/eurheartj/ehae179

[2] Olinic DM, Spinu M, Olinic M et al (2018) Epidemiology of peripheral artery disease in Europe: VAS educational paper. Int Angiol 37(4):327–334. 10.23736/S0392-9590.18.03996-2 29936722 10.23736/S0392-9590.18.03996-2

[3] Kristensen MT, Holm G, Kirketerp-Møller K, Krasheninnikoff M, Gebuhr P (2012) Very low survival rates after non-traumatic lower limb amputation in a consecutive series: what to do? Interact Cardiovasc Thorac Surg 14(5):543–547. 10.1093/ICVTS/IVR075 22298857 10.1093/icvts/ivr075 PMC3329303

[4] Shishehbor MH, Powell RJ, Montero-Baker MF et al (2023) Transcatheter arterialization of deep veins in chronic limb-threatening ischemia. N Engl J Med 388(13):1171–1180. 10.1056/NEJMOA2212754 36988592 10.1056/NEJMoa2212754

[5] Mills JL, Conte MS, Armstrong DG et al (2014) The society for vascular surgery lower extremity threatened limb classification system: risk stratification based on wound, ischemia, and foot infection (WIfI). J Vasc Surg. 10.1016/J.JVS.2013.08.003 24126108 10.1016/j.jvs.2013.08.003

